# The effect of exercise on the risk of metabolic syndrome associated with sleep insufficiency: a cross-sectional study

**DOI:** 10.3389/fcvm.2023.1192241

**Published:** 2023-09-22

**Authors:** Fan-Ya Chou, Te-Fa Chiu, Fen-Wei Huang, Tai-Yi Hsu, Chien-Yu Liu, Chin-Han Lin, Po-Yao Huang, Kuei-Ming Lin, Shih-Hao Wu

**Affiliations:** ^1^ Department of Emergency Medicine, China Medical University Hospital, Taichung, Taiwan; ^2^ College of Medicine, China Medical University, Taichung, Taiwan; ^3^ Department of Public Health, China Medical University, Taichung, Taiwan; ^4^ Department of Bioinformatics and Medical Engineering, Asia University, Taichung, Taiwan

**Keywords:** insomnia, obesity, cardiovascular disease, sleep disturbance, physical activity

## Abstract

**Introduction:**

Sleep disturbance and insufficient sleep have been linked to metabolic syndrome, increasing cardiovascular disease and mortality risk. However, few studies investigate the joint effect of sleep and exercise on metabolic syndrome. We hypothesized that regular exercise can mitigate the exacerbation of metabolic syndrome by sleep insufficiency.

**Objective:**

The aim of this study was to investigate whether exercise can attenuate or eliminate the relationship between sleep insufficiency and metabolic syndrome.

**Method:**

A total of 6,289 adults (mean age = 33.96 years; women: 74.81%) were included in the study, a cross-sectional study conducted based on the results of employee health screening questionnaires and databases from a large healthcare system in central Taiwan. Participants reported sleep insufficiency or not. Self-reported exercise habits were classified into 3 levels: no exercise, exercise <150 min/week, and exercise ≧150 min/week. Multiple logistic regression and sensitivity analyses were conducted to understand the joint associations of sleep patterns and exercise with metabolic syndrome with exposure variables combining sleep duration/disturbances and PA.

**Results:**

Compared with the reference group (sufficient sleep), individuals with sleep insufficiency had a higher risk for metabolic syndrome [adjusted odds ratio (AOR) = 1.40, 95% confidence interval (95% CI): 1.01–1.94, *p* < 0.05] in females aged 40–64 years, but not in other populations. Sleep insufficiency was not associated with the risk of metabolic syndrome among individuals achieving an exercise level of <150 min/week, and in particular among those achieving ≧150 min/week in all populations in our study.

**Conclusion:**

Sleep insufficiency was related to a higher risk of metabolic syndrome in female healthcare staff aged 40–64 years. Being physically active with exercise habits in these individuals, the risk of metabolic syndrome was no longer significant.

## 1. Introduction

Sleep disturbances are common in Chinese healthcare professionals, and their prevalence is much higher than the general population (1). Up to 70 million American adults suffer from sleep problems (2). Insomnia increases the risk of depression, metabolic syndrome, cardiovascular disease, and cancer (3, 4). In addition, sleeping for more than 8 h a day, having difficulty falling asleep, and using sleeping pills increase all-cause mortality, cardiovascular disease mortality, and cancer mortality. Being physically active eliminates these effects (5). Insufficient sleep could lead to a cascade of disorders through endocrine and metabolic changes associated with diabetes (insulin resistance) and weight gain, all of which increase the risk of metabolic syndrome (6–8). Furthermore, modern working patterns, lifestyles and technologies are often not conducive to adequate sleep at times when the internal physiological clock is promoting it (for example, late-night screen time, shift work and nocturnal social activities) (8). In hospital staff, work stress and long hours reduce the time they have for sleep and exercise, putting them at higher risk of developing metabolic syndrome (9). Night shifts increase the prevalence of shift work sleep disorders (10) and exacerbate the situation (11). Physical activity (PA) is beneficial for both mental and physical health. Being physically active not only reduces anxiety and depression, but also improves cardiorespiratory fitness and muscular strength (12, 13). Moreover, it reduces the risk of metabolic syndrome and major cardiovascular disease (4). Both aerobic exercise and resistance training have been proved to decrease the risk of type 2 diabetes (14). PA is also associated with lower all-cause mortality (5, 15). The association between physical activity and sleep is complicated, since exercising late in the evening or night may affect the release of melatonin (3). Aside from this, there is mounting evidence revealing that PA improves both the quality and the quantity of sleep (16–19).

Metabolic syndrome includes central obesity, hypertension, hyperglycemia, and hyperlipidemia (20, 21). Approximately one-third of adults in the United States suffer from metabolic syndrome, and this number is still on the rise (21). Some of the risk factors for metabolic syndrome are obesity, smoking, alcohol consumption, fast food consumption, insomnia, a sedentary lifestyle, and night shifts (9, 22, 23). Metabolic syndrome puts individuals at a higher risk of cardiovascular disease, diabetes, and even death (4, 20, 24). To prevent metabolic syndrome, it is crucial to cultivate a habit of exercising and improve diet and sleep quality (21).

PA has been proven effective in preventing metabolic syndrome and improving the quality of sleep. However, few studies investigate the joint effect of sleep and exercise on metabolic syndrome. Whether it attenuates the detrimental association between sleep insufficiency and metabolic syndrome is still unknown. We hypothesized that regular exercise can mitigate the exacerbation of metabolic syndrome by sleep insufficiency, and aimed to explore the association between self-reported sleep insufficiency and exercising habits with metabolic syndrome.

## 2. Methods

### 2.1. Study population

**Figure 1:**
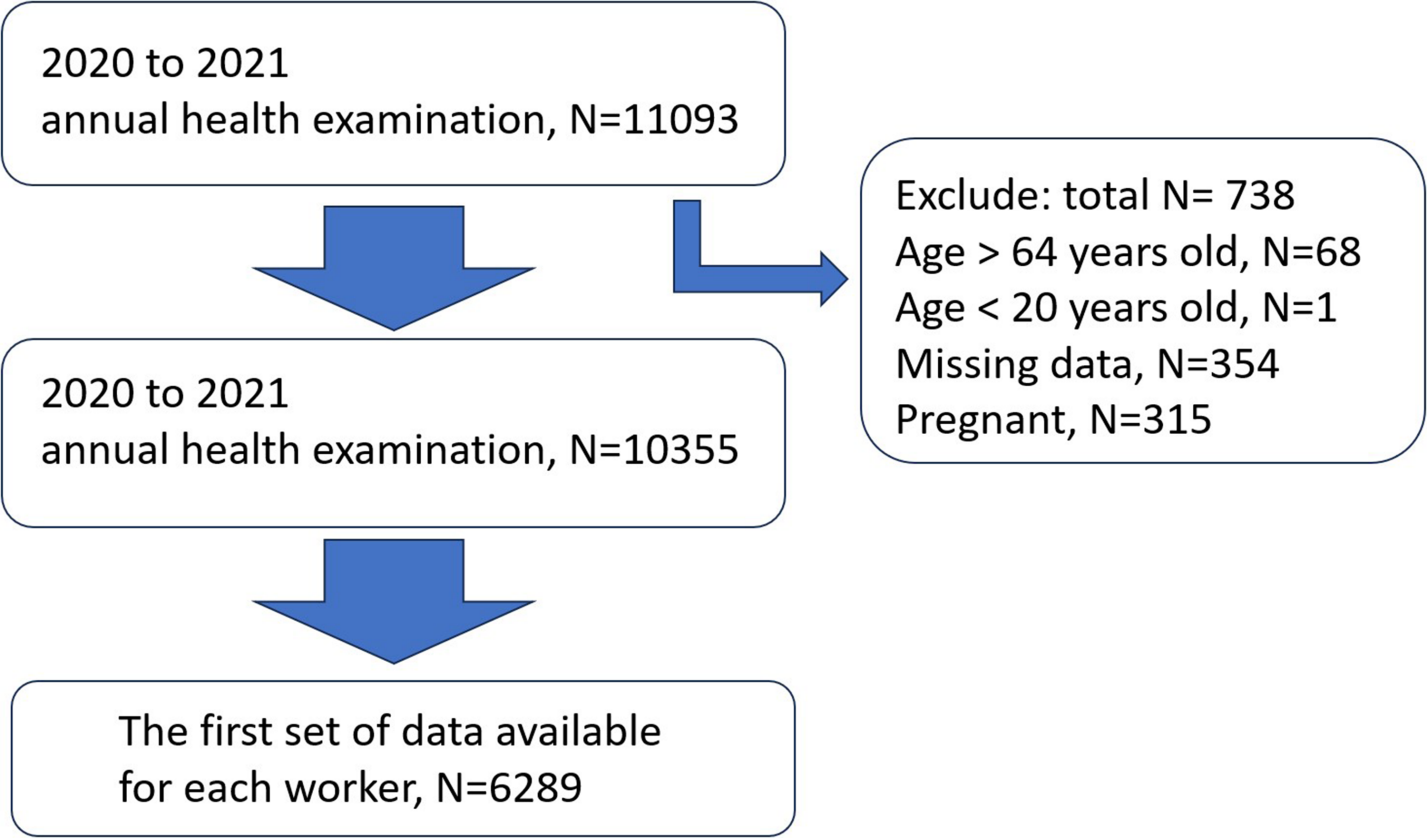
Flowchart of study participant selection from employees of a single medical center.

We carried out a cross-sectional study using the Strengthening the Reporting of Observational studies in Epidemiology (STROBE) guidelines (25) at the China Medical University Hospital (CMUH), Taiwan, from January 2020 to December 2021. According to occupational health regulations in Taiwan, workers across the country are required to undergo annual health screenings, where they provide anthropometric measurements and undergo physical examination and blood tests. We obtained data of 11,093 subjects with informed consent from the information department of the hospital. Participants were excluded if they were not between 20 and 64 years old (*N* = 69), if they did not complete the health screen (with missing data, *N* = 354), and if they were pregnant in the study period (*N* = 315). We chose the first set of data available for each worker (without pregnancy in the case of women workers) during this period. (Figure 1), and they were asked to complete a self-administered questionnaire. The questionnaire included questions about sleep and several lifestyle questions (e.g., smoking, alcohol consumption, sleep, and exercise). The project was approved by the Institutional Review Board of China Medical University Hospital (CMUH110-REC3-205), and all participants provided written informed consent before enrolling in the study.

### 2.2. Data collection

#### 2.2.1. Measures of sleep patterns

Questions of sleep insufficiency attempt to indicate healthy sleep more directly than measures of sleep duration, which may rule out important individual differences in sleep requirements. Altman et al. had demonstrate a study to investigate self-reported sleep duration and self-reported sleep insufficiency as predictors of cardiometabolic health outcomes by telephone interview (26). In this study, participants were asked whether they were getting sufficient sleep through the self-administered questionnaire. Insufficient (Inadequate) sleep means either not enough sleeping time or poor sleep quality. On the basis of the participants’ self-awareness, we categorized them into two groups: One in which the participants got sufficient (adequate) sleep and one in which the participants got insufficient sleep.

#### 2.2.2. Measures of physical activity

The World Health Organization (WHO) suggests that adults aged 18–64 years should do at least 150–300 min of moderate-intensity aerobic PA in a week. In this study, participants were asked three questions concerning physical activity through a modified short International Physical Activity Questionnaire (IPQA) (27, 28): “Do you do exercise?” “How many times do you exercise in a week?” and “How much time do you usually spend doing exercise?” On the basis of the WHO's suggestion, we categorized the participants into three groups: No exercise habits, exercise for less than 150 min per week, and exercise for more than (or exactly) 150 min per week.

#### 2.2.3. Ascertainment of metabolic syndrome

In this study, the diagnostic criteria for metabolic syndrome were the post-2004 revision of the NCEP ATP III diagnostic criteria and the 2005 American Heart Association (AHA) and National Heart, Lung, and Blood Institute (NCLBI) criteria (29, 30). According to the NCEP ATP III definition, metabolic syndrome is present if three or more of the following five criteria are met: (1) In the Asian context, a waist circumference of at least 90 cm in men and 80 cm in women, measured at the top of the iliac crest at the end of a normal expiration; (2) systolic blood pressure of at least 130 mmHg or diastolic blood pressure of at least 85 mmHg, or receiving pharmacologic therapy for hypertension; (3) a triglyceride (TG) level of at least 150 mg/dl or receiving pharmacologic therapy for elevated TG levels; (4) a high-density lipoprotein (HDL) cholesterol level of less than 40 mg/dl in men and 50 mg/dl in women or receiving pharmacologic therapy for reduced HDL cholesterol levels; (5) a fasting glucose level of at least 100 mg/dl or receiving pharmacologic therapy for elevated fasting glucose levels.

#### 2.2.4. Possible confounding factors

We selected covariates *a priori* based on previous literature: sex, age, and job tenure, shift duty categorized as day shift only and other shifts except for day shift (defined as working in the evening from 4:00 p.m. to 0:00 a.m. and in the night from 0:00 a.m. to 8:00 a.m.) (23, 31), personal habits (smoking, alcohol drinking, betel nut chewing, dining out, etc.) (11), and medications (e.g., sleeping pills) (32).

### 2.3. Data analyses

The percentage of each demographic characteristic was presented using frequency distribution. We used the chi-square test to compare the risk of metabolic syndrome between different demographic characteristics. ANOVA was adopted to assess the difference in continuous variables (age, PA hours, etc.) between occupation categories. A *t*-test was used to assess the risk of metabolic syndrome between continuous variables.

Our healthcare population could not represent the general population, so it is not appropriate to use relative risk (RR) (33, 34), besides, the number of cases of sleep insufficiency with metabolic syndrome is relatively small, the odds and risk are similar (34, 35). Therefore, multiple logistic regression was used to assess the relationship between different occupational categories and metabolic syndrome, to determine the risk factors for metabolic syndrome in healthcare staff, and to include interferer correction. The correlation between exercise habits and metabolic syndrome and the correlation between sleep status and metabolic syndrome were analyzed to estimate the adjusted odds ratio (AOR) for the explanatory variables, with a ratio greater than 1 indicating a positive correlation and the opposite indicating a negative correlation. The 95% confidence interval was used to examine the statistical significance of the adjusted odds ratio. We derived a variable combining sleep insufficiency or not and the exercise habits of six groups, where the group with sufficient sleep and the highest exercise amount (equal to or more than 150 min/week) served as the reference group. The adjusted odd ratios of metabolic syndrome for each group were compared to the reference group.

In our analyses, we did not adjust for clinical sleeping disorders (sleep apnea, insomnia, etc.) or mental health due to the small amount of such data in our cohort. To evaluate the robustness of our results and to reduce the possibility of spurious associations due to residual confounding, we conducted a set of sensitivity analyses where we excluded (a) those who suffered from clinical sleep disorders (sleep apnea, insomnia, and other sleep disorders) and (b) those who used psychiatric medications.

For all statistical analyses, the level of significance was set at 0.05. All statistical assessments were two sided and performed with SAS (version 9.4, SAS Institute, Inc., Cary, NC).

## 3. Results

### 3.1. Baseline demographic

Among the 6,289 participants in the core analyses, 712 (11.3%) were diagnosed with metabolic syndrome. Table 1 presents the characteristics of the participants with and without metabolic syndrome. The participants had a mean age of 33.96 ± 9.66 years, with 4,592 (73.02%) aged 20–39 years and 1,697 (26.98%) aged 40–64 years. Among the participants, 4,705 (74.81%) were femal (Table 1).

**Table 1:** Baseline demographics of the participants with and without metabolic syndrome.

Variables	Entire sample	Metabolic syndrome	*p*-value	
	(*n* = 6,289)	Yes (*n* = 712)	No (*n* = 5,577)	
Age (years)	33.96 ± 9.66	40.02 ± 10.32	33.19 ± 9.29	<0.001*
Age				<0.001*
20–40 years	4,592 (73.02)	347 (7.56)	4,245 (92.44)	
40–64 years	1,697 (26.98)	365 (21.51)	1,332 (78.49)	
Sex				<0.001*
Male	1,584 (25.19)	312 (19.70)	1,272 (80.30)	
Female	4,705 (74.81)	400 (8.50)	4,305 (91.50)	
BMI (kg/m^2^)	23.46 ± 4.40	29.71 ± 4.26	22.66 ± 3.73	<0.001
SBP (mmHg)	116.0 ± 13.40	130.4 ± 13.56	114.1 ± 12.20	<0.001
DBP (mmHg)	73.90 ± 11.15	86.24 ± 11.34	72.32 ± 10.09	<0.001
Waist circumference (cm)	75.24 ± 11.62	91.96 ± 9.50	73.10 ± 10.02	<0.001
Glucose (mg/dl)	89.52 ± 14.94	105.4 ± 31.43	87.48 ± 9.43	<0.001
LDL (mg/dl)	108.3 ± 29.60	120.4 ± 33.90	106.8 ± 28.65	<0.001
HDL (mg/dl)	57.84 ± 13.82	42.47 ± 8.44	59.81 ± 13.12	<0.001
Cholesterol (mg/dl)	180.9 ± 33.32	187.1 ± 38.61	180.2 ± 32.50	<0.001
Occupation				<0.001*
Physician	995 (15.82)	148 (14.87)	847 (85.13)	
Nurse	2,961 (47.08)	246 (8.31)	2,715 (91.69)	
Health professional	977 (15.54)	101 (10.34)	876 (89.66)	
Administration staff	1,356 (21.56)	217 (16.00)	1,139 (84.00)	
Job tenure				<0.001*
≤10	4,693 (74.62)	412 (8.78)	4,281 (91.22)	
10–20	1,009 (16.04)	161 (15.96)	848 (84.04)	
>20	587 (9.33)	139 (23.68)	448 (76.32)	
Working hours past 1 month (h/week)	44.81 ± 11.60	45.26 ± 10.17	44.75 ± 11.77	0.215
Working hours past 6 months (h/week)	45.16 ± 11.76	45.46 ± 10.19	45.12 ± 11.95	0.413
Smoking				<0.001*
Yes	231 (3.67)	58 (25.11)	173 (74.89)	
Never	6,058 (96.33)	654 (10.80)	5,404 (89.20)	
Betel nuts				0.008
Yes	27 (0.43)	8 (29.63)	19 (70.37)	
Never	6,262 (99.57)	704 (11.24)	5,558 (88.76)	
Alcohol				0.025*
Yes	1,970 (31.32)	249 (12.64)	1,721 (87.36)	
Never	4,319 (68.68)	463 (10.72)	3,856 (89.28)	
Personal fatigue score	41.05 ± 18.34	40.78 ± 18.21	41.08 ± 18.36	0.673
Work-related fatigue score	40.41 ± 16.06	39.47 ± 15.74	40.53 ± 16.10	0.098
Duty shift				<0.001*
Day shift	3,689 (58.66)	472 (12.79)	3,217 (87.21)	
Other	2,600 (41.34)	240 (9.23)	2,360 (90.77)	
Exercise habits				0.128
None	4,089 (65.02)	487 (11.91)	3,602 (88.09)	
<150 min/week	1,596 (25.38)	165 (10.34)	1,431 (89.66)	
≥150 min/week	604 (9.60)	60 (9.93)	544 (90.07)	
Lack of sleep				0.598
Yes	2,936 (46.68)	339 (11.55)	2,597 (88.45)	
No	3,353 (53.32)	373 (11.12)	2,980 (88.88)	
Meal on time				0.989
Yes	3,199 (50.87)	362 (11.32)	2,837 (88.68)	
No	3,090 (49.13)	350 (11.33)	2,740 (88.67)	
Dining out				0.166
Yes	3,442 (54.73)	407 (11.82)	3,035 (88.18)	
No	2,847 (45.27)	305 (10.71)	2,542 (89.29)	
Dining out frequency (times/day)				0.366
0	2,847 (45.27)	305 (10.71)	2,542 (89.29)	
1	1,266 (20.13)	147 (11.61)	1,119 (88.39)	
2	2,176 (34.60)	260 (11.95)	1,916 (88.05)	
Sedative hypnotic				0.142
Yes	43 (0.68)	8 (18.60)	35 (81.40)	
No	6,246 (99.32)	704 (11.27)	5,542 (88.73)	
Sleep disorder Hx (sleep apnea + insomnia + other sleep disorders)				<0.001*
Yes	211 (3.36)	44 (20.85)	167 (79.15)	
No	6,078 (96.64)	668 (10.99)	5,410 (89.01)	
Sleep apnea Hx				<0.001*
Yes	31 (0.49)	17 (54.84)	14 (45.16)	
No	6,258 (99.51)	695 (11.11)	5,563 (88.89)	
Insomnia Hx				0.158
Yes	170 (2.70)	25 (14.71)	145 (85.29)	
No	6,119 (97.30)	687 (11.23)	5,432 (88.77)	
Other sleep disorders Hx				0.358
Yes	11 (0.17)	2 (18.18)	9 (81.82)	
No	6,278 (99.83)	710 (11.31)	5,568 (88.69)	

Chi-square test. Two-sample *t*-test. Differences between the two groups were analyzed with chi-square tests for categoric variables and independent-samples *t*-tests for continuous variables. Significant variables were then entered into a stepwise backward logistic regression analysis.

* *p* < 0.05.

In all, 2,936 (46.68%) participants considered that they did not get enough sleep, 43 (0.68%) took sleeping pills and 211 (3.36%) had sleep disorders, including 31 (0.49%) with obstructive sleep apnea, 170 (2.70%) with insomnia, and 11 (0.17%) people with other sleep disorders (Table 1).

Of the participants, 4,089 (65.02%) had no exercise habits. Only 604 (9.60%) participants met the WHO criteria for exercising more than 150 min per week (Table 1).

### 3.2. The association between sleep insufficiency and exercise habits and metabolic syndrome

Table 2 presents the association between sleep insufficiency and exercise habits and metabolic syndrome by multiple logistic regression. Supplementary Table S2 presents the results for all covariates used in the model. Compared to the reference group (sufficient sleep), individuals with sleep insufficiency had a higher risk of metabolic syndrome (adjusted odds ratio (AOR) [95% confidence interval (95% CI)] 1.18 (0.99–1.39), *p* = 0.052) (Table 2). In addition, females aged 40–64 years with sleep insufficiency had a statistically significantly increased risk of metabolic syndrome, but the result did not achieve the significance threshold. [AOR (95% CI): 1.40 (1.01–1.94), *p* = 0.044] (Table 3). Therefore, in this healthcare system, perceived sleep insufficiency may be a problem serious enough to increase the risk of metabolic syndrome.

**Table 2:** Risk of metabolic syndrome by sleep and exercise.

	20–64 years old								
	Total			Male			Female		
	AOR	95% CI	*p*-value	AOR	95% CI	*p*-value	AOR	95% CI	*p*-value
Insufficient sleep									
Never	1.00	–	–	1.00	–	–	1.00	–	–
Yes	1.18	0.99–1.39	0.052	1.23	0.94–1.60	0.126	1.15	0.93–1.42	0.195
Exercise habits									
No exercise	1.00	–	–	1.00	–	–	1.00	–	–
<150 min/week	0.66	0.54–0.80	<0.001*	0.70	0.52–0.94	0.016*	0.64	0.48–0.84	0.001*
≥150 min/week	0.54	0.39–0.72	<0.001*	0.59	0.40–0.87	0.008*	0.46	0.27–0.75	0.002*

Confounding factors: Age, sex, occupation, job tenure, shift, smoking, and alcohol consumption.

* *p* < 0.05.

**Table 3:** Risk of metabolic syndrome by sleep and exercise for participants aged between 40 and 64 years.

	40 to 64 years old								
	Total			Male			Female		
	AOR	95% CI	*p*-value	AOR	95% CI	*p*-value	AOR	95% CI	*p*-value
Insufficient sleep									
Never	1.00	–	–	1.00	–	–	1.00	–	–
Yes	1.24	0.97–1.59	0.081	1.06	0.73–1.55	0.741	1.40	1.01–1.94	0.044*
Exercise habits									
No exercise	1.00	–	–	1.00	–	–	1.00	–	–
<150 min/week	0.75	0.57–1.00	0.050	0.82	0.55–1.24	0.356	0.68	0.45–1.01	0.061
≥150 min/week	0.62	0.41–0.91	0.017*	0.67	0.39–1.14	0.147	0.55	0.28–1.01	0.067

Corrected with confounding factors: Age, sex, occupation, job tenure, shift, smoking, and alcohol consumption.

* *p* < 0.05.

PA significantly reduced the risk of metabolic syndrome in active individuals compared with those who did not exercise. Exercising for less than 150 min per week reduced the risk of metabolic syndrome to 66% [AOR (95% CI): 0.66 (0.54–0.80), *p* < 0.001]. Meanwhile, exercising for more than 150 min per week reduced the risk of metabolic syndrome to 54% [AOR (95% CI): 0.54 (0.39–0.72), *p* < 0.001], even more so in women, reducing it to only 46% [AOR (95% CI): 0.46 (0.27–0.75), *p* = 0.002] (Table 2). Therefore, it is clear that exercise can significantly reduce the risk of metabolic syndrome, no matter the amount of exercise.

### 3.3. The association between sleep, exercise, and metabolic syndrome

**Figure 2:**
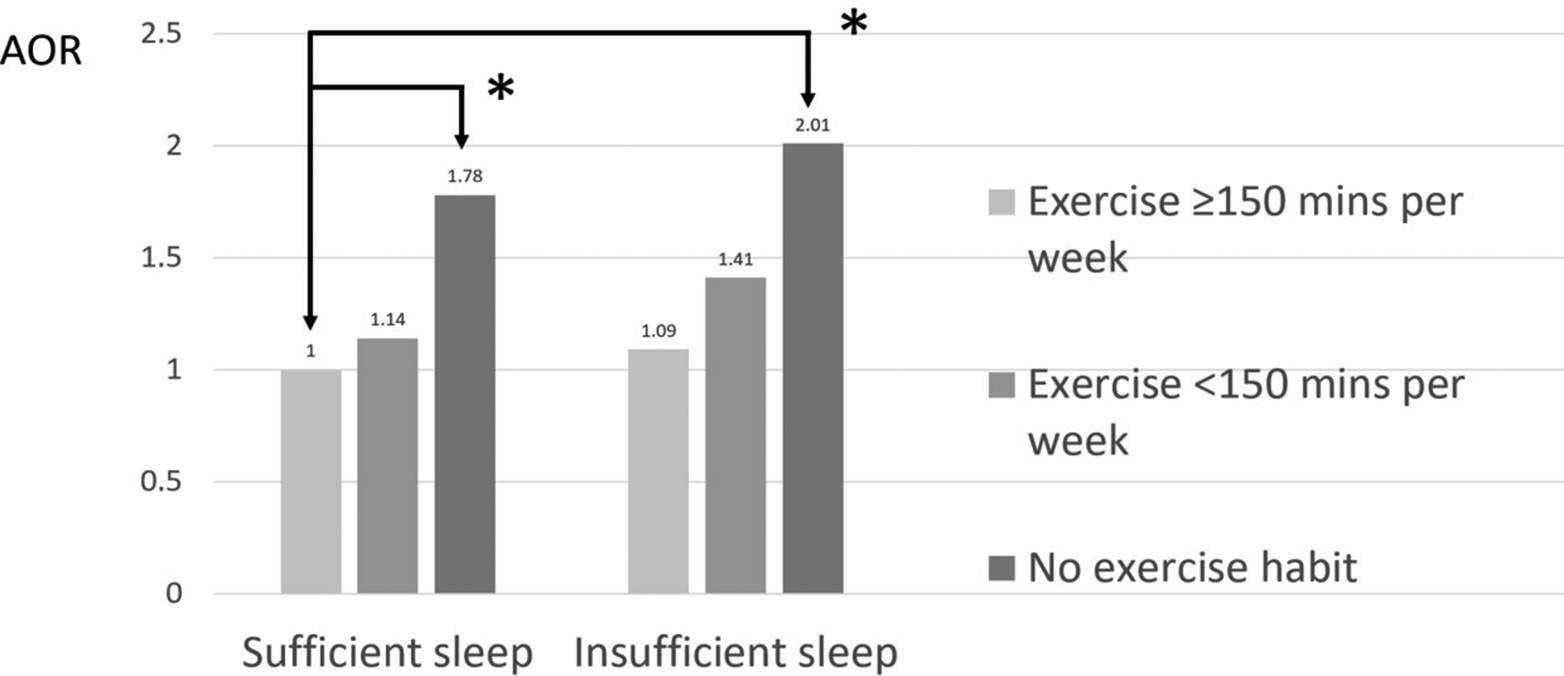
Joint association between sleep insufficiency and exercise habits and metabolic syndrome. *p < 0.05.

The joint association between sleep insufficiency and exercise habits and metabolic syndrome is shown in Table 4. We set the group in which individuals had sufficient sleep and exercised for at least 150 min per week as the reference group. Compared to the reference group, the group in which individuals had insufficient sleep and no exercise habits had a 2.01-fold increased risk of metabolic syndrome [AOR (95% CI): 2.01 (1.37–3.02), *p* < 0.001], while the group in which individuals had insufficient sleep and exercised for less than 150 min per week had a 1.41-fold increased risk of metabolic syndrome [AOR (95% CI): 1.41 (0.92–2.21), *p* = 0.120], and the group in which individuals had insufficient sleep but exercised for at least 150 min per week had a 1.09-fold increased possibility of metabolic syndrome [AOR (95% CI): 1.09 (0.61–1.89), *p* = 0.773] (Table 4 and Figure 2). It can be concluded that with increased exercise, the risk of metabolic syndrome decreases and is no longer statistically significant.

**Table 4:** Joint association between sleep insufficiency and exercise habits and metabolic syndrome.

Variables	All		Excluding participants on sleeping pills or with sleeping disorders	
	AOR (95% CI)	*p*-value	AOR (95% CI)	*p*-value
Sleep insufficiency and exercise habits				
Never and ≥150 min/week	Ref.	–	Ref.	–
Never and <150 min/week	1.14 (0.75–1.77)	0.541	1.20 (0.78–1.88)	0.420
Never and no	1.78 (1.22–2.66)	0.003*	1.82 (1.24–2.76)	0.003*
Yes and ≥150 min/week	1.09 (0.61–1.89)	0.773	1.03 (0.56–1.85)	0.921
Yes and <150 min/week	1.41 (0.92–2.21)	0.120	1.42 (0.91–2.25)	0.126
Yes and no	2.01 (1.37–3.02)	<0.001*	2.00 (1.35–3.04)	<0.001*

Corrected with confounding factors: Age, sex, occupation, job tenure, shift, smoking, and alcohol consumption.

* *p* < 0.05.

**Figure 3:**
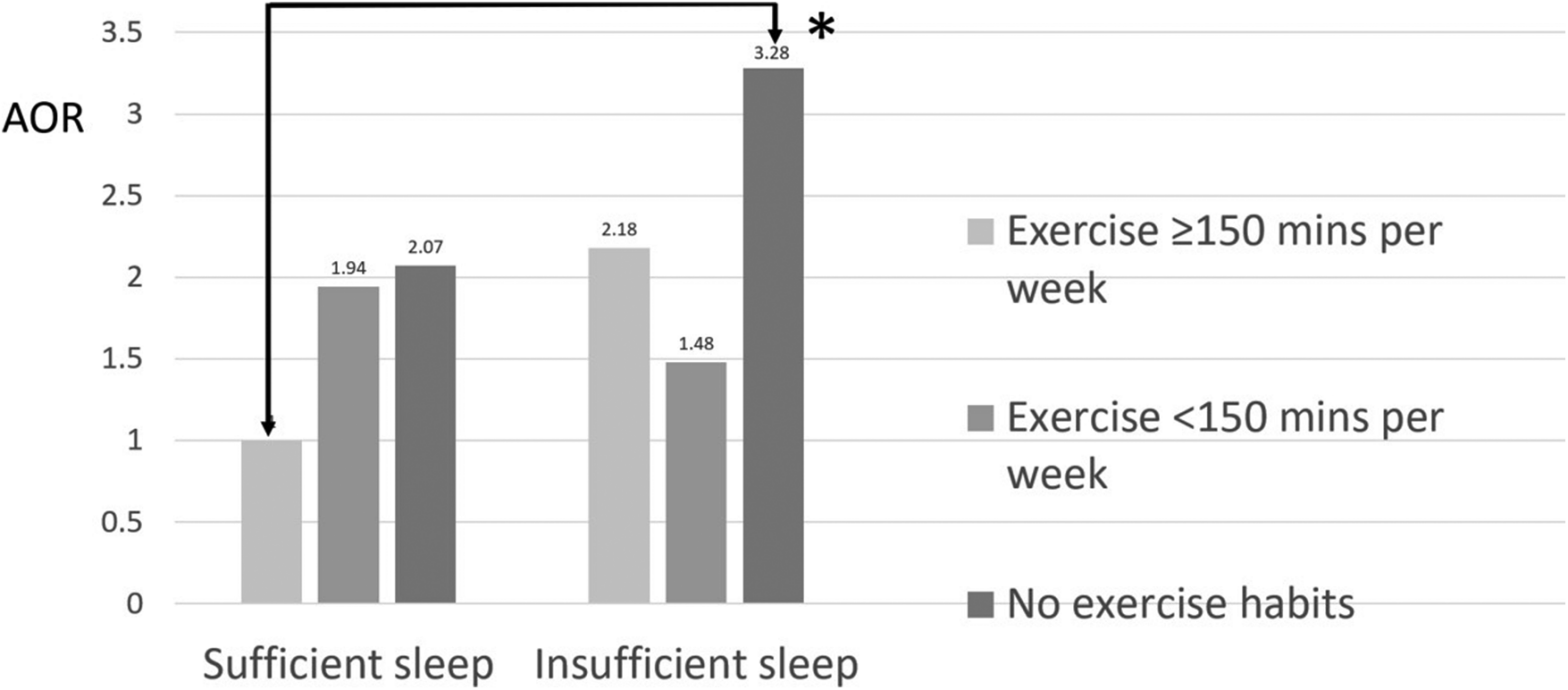
Joint association between sleep insufficiency and exercise habits and metabolic syndrome among females aged 40–64 years. *p < 0.05.

The analysis results for females aged 40–64 years were similar to those for the entire population, as shown in Table 5. We set the group in which women had sufficient sleep and exercised for at least 150 min per week as the reference group. Compared to the reference group, the group in which women had insufficient sleep and no exercise habits had a 3.28-fold increased risk of metabolic syndrome [AOR (95% CI): 3.28 (1.45–8.84), *p* = 0.008], while the group in which women had insufficient sleep and exercised for less than 150 min per week had a 1.48-fold lower risk of metabolic syndrome [AOR (95% CI): 1.48 (0.55–4.44), *p* = 0.456], and the group in which women had insufficient sleep and exercised for at least 150 min per week had a 2.18-fold lower risk of metabolic syndrome [AOR (95% CI): 2.18 (0.62–7.65), *p* = 0.213] (Table 5 and Figure 3). Therefore, sleep insufficiency was related to a higher risk of metabolic syndrome in female healthcare staff aged 40–64 years. Being physically active with exercise habits in these individuals, the risk of metabolic syndrome was no longer significant.

**Table 5:** Joint association between sleep insufficiency and exercise habits and metabolic syndrome among females aged 40–64 years.

Variables	Females aged 40–64 years (*n* = 1,131)		Excluding participants on sleeping pills and with sleeping disorders (*n* = 1,091)	
	AOR (95% CI)	*p*-value	AOR (95% CI)	*p*-value
Sleep insufficiency and exercise habits				
Never and ≥150 min/week	Ref.	–	Ref.	–
Never and <150 min/week	1.94 (0.79–5.49)	0.175	1.89 (0.77–5.37)	0.190
Never and no	2.07 (0.91–5.57)	0.109	2.01 (0.89–5.41)	0.124
Yes and ≥150 min/week	2.18 (0.62–7.65)	0.213	1.95 (0.51–7.11)	0.309
Yes and <150 min/week	1.48 (0.55–4.44)	0.456	1.45 (0.53–4.38)	0.488
Yes and no	3.28 (1.45–8.84)	0.008*	3.17 (1.40–8.54)	0.011*

Confounding factors: Age, sex, occupation, job tenure, shift, smoking, and alcohol consumption.

* *p* < 0.05.

We analyzed the data after removing those participants on sleeping pills and with sleeping disorders, and the sensitivity analysis showed the same results (Table 5).

## 4. Discussion

Our study revealed that the prevalence of metabolic syndrome in the healthcare system at a tertiary medical center in Taiwan was 11.3% and exercise attenuated the risk of metabolic syndrome caused by sleeping insufficiency in females aged 40–64 years.

### 4.1. Baseline demographics

According to our study, the prevalence of metabolic syndrome in a healthcare system at a tertiary medical center in Taiwan was 11.3%, which is far lower than the 33% prevalence in the overall population in the United States (36). This may be related to the fact that nearly three-quarters of the staff in this health system were under the age of 40 years. The prevalence of metabolic syndrome in another healthcare system in Taiwan was 12%, similar to our findings (37).

In our study, 46.7% of the medical staff suffered from sleep insufficiency A meta-analysis in 2021 showed that the prevalence of insomnia among healthcare workers during the COVID-19 pandemic was 42% (38). Similarly, a cross-sectional study conducted in Shanghai, China, in 2022 showed a 55.9% prevalence of poor sleep quality and a 49.2% prevalence of insomnia among healthcare workers vaccinated during the COVID-19 pandemic (39). Although the results are similar to ours, both studies highlight the impact of the COVID-19 pandemic on healthcare workers in terms of stress, which may lead to an increased prevalence of insomnia.

In our results, exercise was not a common habit among medical staff. Although there is plenty of evidence of the positive effects of exercise on health and cognition, only 25% of American adults meet the WHO criteria of weekly exercise amount suggestions (at least 150 min of moderate-intensity aerobic exercise or at least two days of weight training) (40). In contrast, only 15% of Taiwanese adults meet the criteria (5). In our study, only 9.6% of the participants met the standard. It is clear that the amount of exercise Taiwanese adults do is far from enough compared with that of Americans.

### 4.2. The association between sleep insufficiency and exercise habits and metabolic syndrome

Our study revealed that sleep insufficiency has a tendency to increase the risk of metabolic syndrome. Sleep insufficiency significantly increased the risk of metabolic syndrome among females aged 40–64 years. In 2016, a cross-sectional study of 4,197 adults showed that short sleep duration, difficulty falling asleep, and difficulty maintaining sleep are all significantly associated with metabolic syndrome (41). These are all factors of self-reported sleep insufficiency. This can also be described as poor sleep quality, which is related to metabolic syndrome (42). A meta-analysis in 2021 showed that both short and long sleep durations are significantly associated with the growing prevalence of metabolic syndrome (43). In our study, sleep insufficiency tended to increase the risk of metabolic syndrome, but the result did not achieve the significance threshold. This may be related to the lower average age of our study population. Thus, when we isolated the 40–64-year-old age group, we found that sleep insufficiency increased the risk of metabolic syndrome in women.

Regarding exercise habits, we found that, with exercise, the risk of metabolic syndrome was significantly reduced in both men and women, regardless of the amount of exercise. A study by Amirfaiz and Shahril in 2019 showed that the prevalence of metabolic syndrome is positively associated with physical inactivity and sedentary time, while it is negatively associated with the intensity of various physical activities and the number of steps taken (44). A study in 2017 also pointed out that leisure time PA of any amount is better than no PA and that leisure time PA above the current guideline recommendations can further reduce the risk of metabolic syndrome (45). However, in the healthcare system we studied, the population that exercised was relatively small, which may be related to a busy, stressful work life and little free time or to the lack of an exercise environment and motivation.

### 4.3. The association between sleep, exercise, and metabolic syndrome

To the best of our knowledge, our study is the first on the joint effect of sleep and exercise on metabolic syndrome. The aim was to determine whether exercise can eliminate the increased risk of metabolic syndrome caused by sleep insufficiency in healthcare staff.

As per our results, increased exercise was able to alleviate the detrimental relationship between insufficient sleep and metabolic syndrome in females aged 40–64 years. Apparently, the relationship between sleep insufficiency and metabolic syndrome became insignificant as exercise increased. In our whole population, with increased exercise, the risk of metabolic syndrome decreased. However, compared to the reference group, in females aged 40–64 years with sleep insufficiency, the risk of metabolic syndrome increased from 1.48-fold in individuals who exercised for less than 150 min per week (*N* = 123) to 2.18-fold in individuals who exercised for at least 150 min per week (*N* = 38). The plausible possibility is that some individuals who already have metabolic syndrome have a stronger willpower and exercise for at least 150 min per week in order to address their metabolic syndrome. In our data, individuals aged 40–64 years had more population engaged in exercise compared to individuals aged 20–39 years (exercising for at least 150 min per week, 11.90% vs. 8.75%; exercising for less than 150 min per week, 27.88% vs. 24.46%; *p* = 0.084). Among younger females aged 20–39 years, fewer were engaged in exercise. Being unmarried was associated with being highly sedentary (46). In addition, married people may have small children, which is a risk factor for physical inactivity among shift workers (47). This suggests that healthcare staff pay more attention to their health and increase their exercise as they age and have more free time. However, the population engaged in exercise is still far below the US average (40).

Sleep insufficiency by itself did not significantly increase the risk of metabolic syndrome, regardless of the other covariates. However, after adjusting with the other covariates, the sleep insufficiency group had a trend to increase the risk of metabolic syndrome compared to the group without sleep insufficiency, but the result did not achieve the significance threshold [AOR (95% CI): 1.18 (0.99–1.39), *p* = 0.052] (Table 2). In addition, females aged 40–64 years with sleep insufficiency had a significantly increased risk of metabolic syndrome [AOR (95% CI): 1.40 (1.01–1.94), *p* = 0.044] (Table 3). Sleep insufficiency combined with no exercise further increased the risk in all populations [AOR (95% CI): 2.01 (1.37–3.02), *p* < 0.001] (Table 4) and in females aged 40–64 years [AOR (95% CI): 3.28 (1.45–8.84), *p* = 0.008] (Table 5). Therefore, exercise had a greater impact on the risk of metabolic syndrome than sleep.

Our research demonstrated that exercise decreases the risk of metabolic syndrome caused by sleep insufficiency, including short sleep duration or other sleep disturbances. Short sleep duration is related to obesity, inflammation, and cardiovascular diseases (48–50). Sleep disturbances are also a risk factor for depression and dementia (51–53). Both may lead to a decrease in the amount one exercises. However, increasing the amount of exercise is expected to reduce the risk of obesity and cardiovascular disease (54, 55). PA is effective and safe for improving sleep in both healthy and co-morbid populations with sleep disturbance by increasing daily activity levels using a variety of strategies, even low intensity, such as housekeeping, sit-to-stand repetitions, along with encouraging PA through web pages, videos, and self-goal setting apps (19). The positive relationship between exercise and health has been shown to be a powerful defense that counteracts the negative effects of sleep insufficiency.

### 4.4. Strengths and limitations

Our study population of more than 6,000 individuals represents people across healthcare systems. However, our study has some limitations. First, participants were relatively young and tended to have a slightly higher socioeconomic status than the general adult population. However, this lack of representativeness is highly unlikely to have influenced the associations we examined (56). Second, the relatively young age of our population made the relationship between sleep insufficiency and metabolic syndrome even more ambiguous. Among the 40–64-year-olds, the benefits of exercise in preventing or reducing metabolic syndrome became less pronounced with age (Tables 2, 3). However, the detrimental effects of sleep insufficiency on metabolic syndrome (43, 57) and the benefits of exercise in preventing or reducing metabolic syndrome have been well documented in the literature (58). Third, another limitation is the peculiarity of participants’ occupations. Surgeons, physiotherapists, operating room and emergency department nurses are doing physically tough work, standing for hours or even running around, but their work is not considered as “aerobic exercise” and therefore not reported in the survey. Fourth, our data were observational and we cannot rule out the possibility of unmeasured confounding. Moreover, exercise amount and sleep sufficiency were self-reported, which may have introduced recall bias. However, previous research suggests good agreement between sleep self-reports and polysomnography (59). It is also notable that some of the CIs are wide. This may have been due to the small sample size in specific groups (60) (e.g., the group of insufficient sleep without exercise habits and the group of insufficient sleep with exercise at least 150 min per week). Finally, an association does not necessarily equal causation.

## 5. Conclusion

Despite these limitations, this study adds to the literature by showing that sleep insufficiency was related to a higher risk of metabolic syndrome in female healthcare staff aged 40–64 years. Being physically active with exercise habits in these individuals, the risk of metabolic syndrome was no longer significant. Our results suggest that the risk of metabolic syndrome from sleep insufficiency is exacerbated by physical inactivity. These findings provide clinicians with the reassurance that in patients with metabolic syndrome related to sleep insufficiency, even small increases in exercise may have some health benefit and this may be a starting point toward meaningful behavioral change. More longitudinal studies with larger sample sizes in the general population are needed to definitively determine the effect of exercise on the detrimental association between sleep insufficiency and metabolic syndrome and to help the public make positive lifestyle changes by including or increasing PA in daily life.

## Data Availability

The raw data supporting the conclusions of this article will be made available by the authors, without undue reservation.

## References

[1] Qiu D Yu Y Li RQ Li YL Xiao SY. Prevalence of sleep disturbances in Chinese healthcare professionals: a systematic review and meta-analysis. Sleep Med. (2020) 67:258–66. 10.1016/j.sleep.2019.01.047 31040078

[2] Minen MT George A Camacho E Yao L Sahu A Campbell M Assessment of smartphone apps for common neurologic conditions (headache, insomnia, and pain): cross-sectional study. JMIR Mhealth Uhealth. (2022) 10(6):e36761. 10.2196/36761 35727625 PMC9257611

[3] Sejbuk M Mirończuk-Chodakowska I Witkowska AM. Sleep quality: a narrative review on nutrition, stimulants, and physical activity as important factors. Nutrients. (2022) 14(9):1912. PMID: ; PMCID: . 10.3390/nu14091912 35565879 PMC9103473

[4] Chen LJ Lai YJ Sun WJ Fox KR Chu D Ku PW. Associations of exercise, sedentary time and insomnia with metabolic syndrome in Taiwanese older adults: a 1-year follow-up study. Endocr Res. (2015) 40(4):220–6. 10.3109/07435800.2015.1020547 26167672

[5] Chen L-J Hamer M Lai Y-J Huang B-H Ku P-W Stamatakis E. Can physical activity eliminate the mortality risk associated with poor sleep? A 15-year follow-up of 341,248 MJ cohort participants. J Sport Health Sci. (2022) 11(5):596–604. 10.1016/j.jshs.2021.03.001 33713846 PMC9532590

[6] Duan D Kim LJ Jun JC Polotsky VY. Connecting insufficient sleep and insomnia with metabolic dysfunction. Ann N Y Acad Sci. (2023) 1519(1):94–117. 10.1111/nyas.14926 36373239 PMC9839511

[7] Bass J Turek FW. Sleepless in America: a pathway to obesity and the metabolic syndrome? Arch Intern Med. (2005) 165(1):15–6. 10.1001/archinte.165.1.15 15642868

[8] Chaput J-P McHill AW Cox RC Broussard JL Dutil C da Costa BGG The role of insufficient sleep and circadian misalignment in obesity. Nat Rev Endocrinol. (2023) 19(2):82–97. 10.1038/s41574-022-00747-7 36280789 PMC9590398

[9] Tsai H-J Tsou M-T. Age, sex, and profession difference among health care workers with burnout and metabolic syndrome in Taiwan tertiary hospital—a cross-section study. Front Med (Lausanne). (2022) 9:854403. PMID: ; PMCID: . 10.3389/fmed.2022.854403 35492349 PMC9048413

[10] Vanttola P Puttonen S Karhula K Oksanen T Härmä M. Prevalence of shift work disorder among hospital personnel: a cross-sectional study using objective working hour data. J Sleep Res. (2020) 29(3):e12906. 10.1111/jsr.12906 31410909

[11] Pietroiusti A Neri A Somma G Coppeta L Iavicoli I Bergamaschi A Incidence of metabolic syndrome among night-shift healthcare workers. Occup Environ Med. (2010) 67(1):54–7. 10.1136/oem.2009.046797 19737731

[12] Chi G Wang L. The association of sports participation with depressive symptoms and anxiety disorder in adolescents. Front Public Health. (2022) 10:860994. PMID: ; PMCID: . 10.3389/fpubh.2022.860994 35719630 PMC9203890

[13] Khalafi M Sakhaei MH Rosenkranz SK Symonds ME. Impact of concurrent training versus aerobic or resistance training on cardiorespiratory fitness and muscular strength in middle-aged to older adults: a systematic review and meta-analysis. Physiol Behav. (2022) 254:113888. 10.1016/j.physbeh.2022.113888 35728627

[14] Warburton DER Nicol CW Bredin SSD. Health benefits of physical activity: the evidence. Can Med Assoc J. (2006) 174(6):801–9. 10.1503/cmaj.051351 16534088 PMC1402378

[15] Lear SA Hu W Rangarajan S Gasevic D Leong D Iqbal R The effect of physical activity on mortality and cardiovascular disease in 130,000 people from 17 high-income, middle-income, and low-income countries: the PURE study. Lancet. (2017) 390(10113):2643–54. 10.1016/S0140-6736(17)31634-3 28943267

[16] Banno M Harada Y Taniguchi M Tobita R Tsujimoto H Tsujimoto Y Exercise can improve sleep quality: a systematic review and meta-analysis. PeerJ. (2018) 6:e5172. 10.7717/peerj.5172 30018855 PMC6045928

[17] Dolezal BA Neufeld EV Boland DM Martin JL Cooper CB. Interrelationship between sleep and exercise: a systematic review. Adv Prev Med. (2017) 2017:1364387. 10.1155/2017/1364387 28458924 PMC5385214

[18] Kredlow MA Capozzoli MC Hearon BA Calkins AW Otto MW. The effects of physical activity on sleep: a meta-analytic review. J Behav Med. (2015) 38(3):427–49. 10.1007/s10865-015-9617-6 25596964

[19] Huang H-H Stubbs B Chen L-J Ku P-W Hsu T-Y Lin C-W The effect of physical activity on sleep disturbance in various populations: a scoping review of randomized clinical trials. Int J Behav Nutr Phys Act. (2023) 20(1):44. 10.1186/s12966-023-01449-7 37069626 PMC10107572

[20] Huang PL. A comprehensive definition for metabolic syndrome. Dis Model Mech. (2009) 2(5–6):231–7. 10.1242/dmm.001180 19407331 PMC2675814

[21] Saklayen MG. The global epidemic of the metabolic syndrome. Curr Hypertens Rep. (2018) 20(2):12. 10.1007/s11906-018-0812-z 29480368 PMC5866840

[22] Cheng W-J Liu C-S Hu K-C Cheng Y-F Karhula K Härmä M. Night shift work and the risk of metabolic syndrome: findings from an 8-year hospital cohort. PLoS One. (2021) 16(12):e0261349. PMID: ; PMCID: . 10.1371/journal.pone.0261349 34898652 PMC8668137

[23] Sooriyaarachchi P Jayawardena R Pavey T King NA. Shift work and the risk for metabolic syndrome among healthcare workers: a systematic review and meta-analysis. Obes Rev. (2022) 23(10):e13489. 10.1111/obr.13489 35734805 PMC9539605

[24] Grabia M Markiewicz-Żukowska R Socha K Polkowska A Zasim A Boruch K Prevalence of metabolic syndrome in relation to cardiovascular biomarkers and dietary factors among adolescents with type 1 diabetes mellitus. Nutrients. (2022) 14(12):2435. PMID: ; PMCID: . 10.3390/nu14122435 35745165 PMC9228781

[25] Cuschieri S. The STROBE guidelines. Saudi J Anaesth. (2019) 13(Suppl 1):S31–4. 10.4103/sja.SJA_543_18 30930717 PMC6398292

[26] Altman NG Izci-Balserak B Schopfer E Jackson N Rattanaumpawan P Gehrman PR Sleep duration versus sleep insufficiency as predictors of cardiometabolic health outcomes. Sleep Med. (2012) 13(10):1261–70. 10.1016/j.sleep.2012.08.005 23141932 PMC3527631

[27] Craig CL Marshall AL Sjöström M Bauman AE Booth ML Ainsworth BE International physical activity questionnaire: 12-country reliability and validity. Med Sci Sports Exerc. (2003) 35(8):1381–95. 10.1249/01.MSS.0000078924.61453.FB 12900694

[28] Ren YJ Su M Liu QM Tan YY Du YK Li LM Lyu J. Validation of the simplified Chinese-character version of the international physical activity questionnaire-long form in urban community-dwelling adults: a cross-sectional study in Hangzhou, China. Biomed Environ Sci*.* (2017) 30(4):255–63. PMID: . 10.3967/bes2017.035 28494835

[29] Grundy SM Brewer HB Jr Cleeman JI Smith SC Jr Lenfant C. Definition of metabolic syndrome: report of the national heart, lung, and blood institute/American heart association conference on scientific issues related to definition. Circulation. (2004) 109(3):433–8. 10.1161/01.CIR.0000111245.75752.C6 14744958

[30] Grundy SM Cleeman JI Daniels SR Donato KA Eckel RH Franklin BA Diagnosis and management of the metabolic syndrome: an American heart association/national heart, lung, and blood institute scientific statement. Circulation. (2005) 112(17):2735–52. 10.1161/CIRCULATIONAHA.105.169404 16157765

[31] Stevens RG Hansen J Costa G Haus E Kauppinen T Aronson KJ Considerations of circadian impact for defining “shift work” in cancer studies: IARC working group report. Occup Environ Med. (2011) 68(2):154–62. 10.1136/oem.2009.053512 20962033

[32] Izumida T Nakamura Y Sato Y Ishikawa S. The association between sleeping pill use and metabolic syndrome in an apparently healthy population in Japan: JMS-II cohort study. J Epidemiol. (2022) 32(3):145–50. 10.2188/jea.JE20200361 33162423 PMC8824657

[33] Tenny S Hoffman MR. Relative Risk. In: StatPearls. Treasure Island (FL): StatPearls Publishing LLC (2023).

[34] Ranganathan P Aggarwal R Pramesh CS. Common pitfalls in statistical analysis: odds versus risk. Perspect Clin Res (2015) 6(4):222–4. 10.4103/2229-3485.167092 26623395 PMC4640017

[35] Sedgwick P. Relative risks versus odds ratios. Br Med J. (2014) 348:g1407. 10.1136/bmj.g1407

[36] Hirode G Wong RJ. Trends in the prevalence of metabolic syndrome in the United States, 2011–2016. JAMA. (2020) 323(24):2526–8. 10.1001/jama.2020.4501 32573660 PMC7312413

[37] Yeh WC Chuang HH Lu MC Tzeng IS Chen JY. Prevalence of metabolic syndrome among employees of a Taiwanese hospital varies according to profession. Medicine (Baltimore). (2018) 97(31):e11664. 10.1097/MD.0000000000011664 30075556 PMC6081057

[38] Aymerich C Pedruzo B Pérez JL Laborda M Herrero J Blanco J COVID-19 pandemic effects on health worker’s mental health: systematic review and meta-analysis. Eur Psychiatry. (2022) 65(1):e10. 10.1192/j.eurpsy.2022.1 35060458 PMC8828390

[39] Wang P Shen X Jiang Y Wu L Shen J Nie X Psychological and sleep disturbances among first-line, second-line, and at home medical staff during the COVID-19 pandemic in Shanghai, China. Front Public Health. (2022) 10:1006610. PMID: ; PMCID: . 10.3389/fpubh.2022.1006610 36420001 PMC9677109

[40] Hyde ET Whitfield GP Omura JD Fulton JE Carlson SA. Trends in meeting the physical activity guidelines: muscle-strengthening alone and combined with aerobic activity, United States, 1998–2018. J Phys Act Health. (2021) 18(S1):S37–44. 10.1123/jpah.2021-0077 34465652 PMC11000248

[41] Lin S-C Sun C-A You S-L Hwang L-C Liang C-Y Yang T The link of self-reported insomnia symptoms and sleep duration with metabolic syndrome: a Chinese population-based study. Sleep. (2016) 39(6):1261–6. 10.5665/sleep.5848 27070137 PMC4863215

[42] Koren D Dumin M Gozal D. Role of sleep quality in the metabolic syndrome. Diabetes Metab Syndr Obes. (2016) 9:281–310. 10.2147/DMSO.S95120 27601926 PMC5003523

[43] Xie J Li Y Zhang YJ Vgontzas AN Basta M Chen BX Sleep duration and metabolic syndrome: an updated systematic review and meta-analysis. Sleep Med Rev. (2021) 59:101451. 10.1016/j.smrv.2021.101451 33618187

[44] Amirfaiz S Shahril MR. Objectively measured physical activity, sedentary behavior, and metabolic syndrome in adults: systematic review of observational evidence. Metab Syndr Relat Disord. (2019) 17(1):1–21. 10.1089/met.2018.0032 30272527

[45] Zhang D Liu X Liu Y Sun X Wang B Ren Y Leisure-time physical activity and incident metabolic syndrome: a systematic review and dose-response meta-analysis of cohort studies. Metab Clin Exp. (2017) 75:36–44. 10.1016/j.metabol.2017.08.001 28927737

[46] Kitayama A Koohsari MJ Ishii K Shibata A Oka K. Sedentary time in a nationally representative sample of adults in Japan: prevalence and sociodemographic correlates. Prev Med Rep. (2021) 23:101439. 10.1016/j.pmedr.2021.101439 34178590 PMC8214138

[47] Cheng WJ Härmä M Ropponen A Karhula K Koskinen A Oksanen T. Shift work and physical inactivity: findings from the Finnish public sector study with objective working hour data. Scand J Work Environ Health. (2020) 46(3):293–301. 10.5271/sjweh.3868 31788701

[48] Itani O Jike M Watanabe N Kaneita Y. Short sleep duration and health outcomes: a systematic review, meta-analysis, and meta-regression. Sleep Med. (2017) 32:246–56. 10.1016/j.sleep.2016.08.006 27743803

[49] Gao C Guo J Gong TT Lv JL Li XY Liu FH Sleep duration/quality with health outcomes: an umbrella review of meta-analyses of prospective studies. Front Med (Lausanne). (2022) 8:813943. PMID: ; PMCID: . 10.3389/fmed.2021.813943 35127769 PMC8811149

[50] Irwin MR Olmstead R Carroll JE Disturbance S Duration S. And inflammation: a systematic review and meta-analysis of cohort studies and experimental sleep deprivation. Biol Psychiatry. (2016) 80(1):40–52. 10.1016/j.biopsych.2015.05.014 26140821 PMC4666828

[51] Irwin MR Vitiello MV. Implications of sleep disturbance and inflammation for Alzheimer’s disease dementia. Lancet Neurology. (2019) 18(3):296–306. 10.1016/S1474-4422(18)30450-2 30661858

[52] Shi L Chen SJ Ma MY Bao YP Han Y Wang YM Sleep disturbances increase the risk of dementia: a systematic review and meta-analysis. Sleep Med Rev. (2018) 40:4–16. 10.1016/j.smrv.2017.06.010 28890168

[53] Riemann D Krone LB Wulff K Nissen C. Sleep, insomnia, and depression. Neuropsychopharmacology. (2020) 45(1):74–89. 10.1038/s41386-019-0411-y 31071719 PMC6879516

[54] Ribeiro FM Silva MA Lyssa V Marques G Lima HK Franco OL The molecular signaling of exercise and obesity in the microbiota-gut-brain axis. Front Endocrinol (Lausanne). (2022) 13:927170. PMID: ; PMCID: . 10.3389/fendo.2022.927170 35966101 PMC9365995

[55] D'Ascenzi F Cavigli L Pagliaro A Focardi M Valente S Cameli M Clinician approach to cardiopulmonary exercise testing for exercise prescription in patients at risk of and with cardiovascular disease. Br J Sports Med. (2022) 56(20):1180. 10.1136/bjsports-2021-105261 35680397

[56] Batty GD Gale CR Kivimäki M Deary IJ Bell S. Comparison of risk factor associations in UK biobank against representative, general population based studies with conventional response rates: prospective cohort study and individual participant meta-analysis. Br Med J. (2020) 368:m131. 10.1136/bmj.m131 32051121 PMC7190071

[57] Che TT Yan C Tian DY Zhang X Liu XJ Wu ZM. The association between sleep and metabolic syndrome: a systematic review and meta-analysis. Front Endocrinol (Lausanne). (2021) 12:773646. PMID: ; PMCID: . 10.3389/fendo.2021.773646 34867820 PMC8640251

[58] Myers J Kokkinos P Nyelin E Activity P. Cardiorespiratory fitness, and the metabolic syndrome. Nutrients. (2019) 11(7):1652. PMID: ; PMCID: . 10.3390/nu11071652 31331009 PMC6683051

[59] Zinkhan M Berger K Hense S Nagel M Obst A Koch B Agreement of different methods for assessing sleep characteristics: a comparison of two actigraphs, wrist and hip placement, and self-report with polysomnography. Sleep Med. (2014) 15(9):1107–14. 10.1016/j.sleep.2014.04.015 25018025

[60] Tzeng IS. To handle the inflation of odds ratios in a retrospective study with a profile penalized log-likelihood approach. J Clin Lab Anal. (2021) 35(7):e23849. 10.1002/jcla.23849 34043251 PMC8274998

