# Supplementary material for: The effect of exercise on the risk of metabolic syndrome associated with sleep insufficiency: a cross-sectional study
Source: Front Cardiovasc Med. 2023 Sep 22;10:1192241. doi: 10.3389/fcvm.2023.1192241 (PMC10556234; doi:10.3389/fcvm.2023.1192241)
Supplement: Supplementary file 1 [file Table1.docx]

Supplementary Material

The Effect of Exercise on the Risk of Metabolic Syndrome Associated with Sleep Insufficiency: A Cross-sectional Study

**Fan-Ya Chou ^#^, Te-Fa Chiu^#^, Fen-Wei Huang, Tai-Yi Hsu, Chien-Yu Liu, Chin-Han Lin, Po-Yao Huang, Kuei-Ming Lin, and Shih-Hao Wu ***

*** Correspondence: Shih-Hao Wu** Corresponding Author: ambertwu@gmail.com

| Table S1. Comparison between different occupations | | | | | |
| --- | --- | --- | --- | --- | --- |
| Variables | Occupation | | | | p-value |
|  | Physician  (n=995) | Nurse  (n=2961) | Health professional (n=977) | Administration staff (n=1356) |  |
| Age |  |  |  |  | <0.001* |
| 20-40 | 645(14.05) | 2466(53.70) | 666(14.50) | 815(17.75) |  |
| 40-64 | 350(20.62) | 495(29.17) | 311(18.33) | 541(31.88) |  |
| Gender |  |  |  |  | <0.001* |
| Male | 687(43.37) | 187(11.81) | 275(17.36) | 435(27.46) |  |
| Female | 308(6.55) | 2774(58.96) | 702(14.92) | 921(19.57) |  |
| Job tenure |  |  |  |  | <0.001* |
| <=10 | 678(14.45) | 2378(50.67) | 676(14.40) | 961(20.48) |  |
| >10 | 317(19.86) | 583(36.53) | 301(18.86) | 395(24.75) |  |
| Duty shift |  |  |  |  | <0.001* |
| Day shift | 716(19.41) | 1246(33.78) | 697(18.89) | 1030(27.92) |  |
| Others | 279(10.73) | 1715(65.96) | 280(10.77) | 326(12.54) |  |
| Exercise habits (min) |  |  |  |  | <0.001* |
| No | 459(11.23) | 2142(52.38) | 589(14.40) | 899(21.99) |  |
| <150 | 385(24.12) | 601(37.66) | 286(17.92) | 324(20.30) |  |
| >=150 | 151(25.00) | 218(36.09) | 102(16.89) | 133(22.02) |  |
| Lack of sleep |  |  |  |  | <0.001* |
| Yes | 524(17.85) | 1472(50.14) | 449(15.29) | 491(16.72) |  |
| No | 471(14.05) | 1489(44.41) | 528(15.75) | 865(25.80) |  |

*: *p value* < 0.05

|  | 20 to 64 years old | | | | | | | | |
| --- | --- | --- | --- | --- | --- | --- | --- | --- | --- |
|  | Total | | | Male | | | Female | | |
|  | AOR | 95% CI | *p value* | AOR | 95% CI | *p value* | AOR | 95% CI | *p value* |
| Age | 1.06 | 1.05-1.08 | *<0.001** | 1.06 | 1.04-1.08 | *<0.001** | 1.07 | 1.05-1.08 | *<0.001** |
| Sex |  |  |  |  |  |  |  |  |  |
| Female | 1.00 | － | － |  |  |  |  |  |  |
| Male | 2.58 | 2.09-3.18 | *<0.001** |  |  |  |  |  |  |
| Occupation |  |  |  |  |  |  |  |  |  |
| Administration staff | 1.00 | － | － | 1.00 | － | － | 1.00 | － | － |
| Health professional | 0.71 | 0.55-0.93 | *0.012** | 0.76 | 0.50-1.14 | *0.195* | 0.67 | 0.47-0.94 | *0.022** |
| Nurse | 0.99 | 0.79-1.25 | *0.941* | 1.18 | 0.71-1.93 | *0.511* | 0.91 | 0.70-1.19 | *0.478* |
| Physician | 0.71 | 0.55-0.92 | *0.009** | 0.86 | 0.63-1.19 | *0.366* | 0.39 | 0.20-0.70 | *0.002** |
| Job tenure |  |  |  |  |  |  |  |  |  |
| ≦10 | 1.00 | － | － | 1.00 | － | － | 1.00 | － | － |
| ＞10 | 1.00 | 0.80-1.24 | *0.993* | 0.91 | 0.64-1.28 | *0.592* | 1.04 | 0.79-1.38 | *0.761* |
| Shift |  |  |  |  |  |  |  |  |  |
| Others | 1.00 | － | － | 1.00 | － | － | 1.00 | － | － |
| Day shift | 1.09 | 0.91-1.31 | *0.339* | 1.04 | 0.78-1.39 | *0.804* | 1.12 | 0.89-1.42 | *0.325* |
| Smoking |  |  |  |  |  |  |  |  |  |
| Never | 1.00 | － | － | 1.00 | － | － | 1.00 | － | － |
| Yes | 1.72 | 1.20-2.44 | *0.002** | 1.64 | 1.08-2.45 | *0.017** | 2.19 | 1.05-4.19 | *0.024** |
| Alcohol |  |  |  |  |  |  |  |  |  |
| Never | 1.00 | － | － | 1.00 | － | － | 1.00 | － | － |
| Yes | 0.99 | 0.82-1.18 | *0.907* | 1.01 | 0.77-1.32 | *0.938* | 0.99 | 0.77-1.25 | *0.908* |

Table S2 . Logistic regression for all covariates entered in the model

*: *p value* < 0.05

|  | 40 to 64 years old | | | | | | | | |
| --- | --- | --- | --- | --- | --- | --- | --- | --- | --- |
|  | Total | | | Male | | | Female | | |
|  | AOR | 95% CI | *p value* | AOR | 95% CI | *p value* | AOR | 95% CI | *p value* |
| Age | 1.04 | 1.02-1.06 | *<0.001** | 1.01 | 0.98-1.04 | *0.393* | 1.06 | 1.03-1.09 | *<0.001** |
| Sex |  |  |  |  |  |  |  |  |  |
| Female | 1.00 | － | － |  |  |  |  |  |  |
| Male | 2.32 | 1.70-3.17 | *<0.001** |  |  |  |  |  |  |
| Occupation |  |  |  |  |  |  |  |  |  |
| Administration staff | 1.00 | － | － | 1.00 | － | － | 1.00 | － | － |
| Health professional | 0.71 | 0.49-1.03 | *0.072* | 0.59 | 0.31-1.10 | *0.107* | 0.76 | 0.47-1.20 | *0.242* |
| Nurse | 1.04 | 0.74-1.47 | *0.808* | 1.68 | 0.61-4.46 | *0.299* | 0.97 | 0.66-1.43 | *0.893* |
| Physician | 1.00 | 0.70-1.41 | *0.993* | 1.32 | 0.87-2.02 | *0.201* | 0.47 | 0.17-1.07 | *0.098* |
| Job tenure |  |  |  |  |  |  |  |  |  |
| ≦10 | 1.00 | － | － | 1.00 | － | － | 1.00 | － | － |
| ＞10 | 0.91 | 0.70-1.19 | *0.480* | 0.67 | 0.45-1.01 | *0.055* | 1.14 | 0.79-1.67 | *0.487* |
| Shift |  |  |  |  |  |  |  |  |  |
| Others | 1.00 | － | － | 1.00 | － | － | 1.00 | － | － |
| Day shift | 1.14 | 0.87-1.52 | *0.347* | 1.02 | 0.67-1.56 | *0.930* | 1.21 | 0.83-1.80 | *0.330* |
| Smoking |  |  |  |  |  |  |  |  |  |
| Never | 1.00 | － | － | 1.00 | － | － | 1.00 | － | － |
| Yes | 1.53 | 0.92-2.51 | *0.096* | 1.54 | 0.88-2.67 | *0.128* | 1.63 | 0.35-5.68 | *0.477* |
| Alcohol |  |  |  |  |  |  |  |  |  |
| Never | 1.00 | － | － | 1.00 | － | － | 1.00 | － | － |
| Yes | 1.03 | 0.79-1.34 | *0.818* | 1.15 | 0.79-1.67 | *0.474* | 0.96 | 0.65-1.40 | *0.842* |

Table S3 . Logistic regression for all covariates entered in the model for 40 to 64 years old population.

*: *p value* < 0.05
